# Understanding the consumption of folic acid during preconception, among Pakistani, Bangladeshi and white British mothers in Luton, UK: a qualitative study

**DOI:** 10.1186/s12884-018-1884-0

**Published:** 2018-06-15

**Authors:** Rebecca Garcia, Nasreen Ali, Malcolm Griffiths, Gurch Randhawa

**Affiliations:** 1 The School of Healthcare Practice, University of Bedfordshire, Putteridge Bury, Hitchin Road, Luton, Luton, LU2 8LE England; 2 The Institute For Health Research, University of Bedfordshire, Putteridge Bury, Hitchin Road, Luton, Luton, LU2 8LE England; 3 Luton & Dunstable University Hospital NHS Foundation Trust, Lewsey Rd, Luton, LU4 0DZ England

**Keywords:** Folic acid, Neural tube defects, South Asian, Pakistani, Indian Bangladeshi white British

## Abstract

**Background:**

To review the similarities and differences in Pakistani, Bangladeshi and White British mothers health beliefs (attitudes, knowledge and perceptions) and health behaviour regarding their consumption of folic acid pre-conception, to reduce the risk of neural tube defects.

**Methods:**

Our study used a descriptive qualitative research approach, implementing face-to-face focus group discussions with Pakistani, Bangladeshi or White British mothers (normal birth outcomes and mothers with poor birth outcomes) and semi-structured interviews or focus groups with service providers using semi-structured topic guides. This method is well suited for under researched areas where in-depth information is sought.

There were three sample groups:

1. Pakistani, Bangladeshi and White British mothers with normal birth outcomes (delivery after 37 weeks of gestation, in the preceding 6 to 24 months, weighing 2500 g and living within a specified postcode area in Luton, UK).
2. Pakistani Bangladeshi and white British bereaved mothers who had suffered a perinatal mortality (preceding 6 to 24 months, residing within a specificied postcode area).
3. Healthcare professionals working on the local maternity care pathway (i.e. services providing preconception, antenatal, antepartum and postpartum care).

Transcribed discussions were analysed using the Framework Analysis approach.

**Results:**

The majority of mothers in this sample did not understand the benefits or optimal time to take folic acid pre-conception. Conversely, healthcare professionals believed the majority of women did consume folic acid, prior to conception.

**Conclusions:**

There is a need to increase public health awareness of the optimal time and subsequent benefits for taking folic acid, to prevent neural tube defects.

**Electronic supplementary material:**

The online version of this article (10.1186/s12884-018-1884-0) contains supplementary material, which is available to authorized users.

## Background

Folic acid consumption pre-conception is necessary for optimal embryogenesis [1, 2]; the benefits are well documented and include reducing the incidence of neural tube defect (NTD) [3–7] in addition to other structural congenital anomalies [8]. NTD are a significant congenital anomaly which account for the second leading cause of infant mortality and also results in high rates of morbidity [9]. Current guidance in the United Kingdom (UK) idenitifies certain women at higher risk of having an infant with NTDs; for instance, a family history of NTD, medical conditions such as diabetes, or thalassemia or women identified with a body mass index (BMI) of > 30 kg/m^2^ [10].

Public health surveillance has shown that there is an increasing prevalence of NTD [2], while trends for consumption of folic acid have decreased [11, 12]. Estimates of consumption rates range from 10.4–35% of pregnancies, with estimates for South Asian women (i.e. Indian, Pakistani, Bangladeshi or Sri Lankan) are even lower (20%) [11, 13, 14]. Figures have also shown there is an ethnic variation to the incidence of congenital anomalies; the leading cause of death in South Asian infants death being congenital anomalies, however in White infants it related to fetal maturity [15]. Furthermore, it has been estimated that only half of all pregnancies are actually planned [16], suggesting that increasing trends of NTD and high numbers of unplanned pregnancies indicate that many women have poor health literacy on the benefits of folic acid consumption before pregnancy occurs. In the U.K., folic acid may be prescribed at a higher dose (5 mg) by General Practitioners under certain circumstances (e.g. risk or previous history of NTD, consumption of anti-epilepsy medication, history of coeliac, diabetes mellitus, sickle cell anaemia or thalassemia, or obesity, [10]), alternatively women can readily purchase folic acid supplements (400mcg) from pharmacies.

Maternal attitudes, knowledge and perceptions have been found to be more indicative of birth outcome than deprivation [17], with a recent study providing evidence demonstrative of various barriers to consuming folic acid, including poor knowledge, unplanned pregnancy and poor memory as a result of leading complex lives [18]. Moreover, inadvertent disadvantage may occur with (some) mothers, as a consequence of lower educational attainment, reduced health literacy, language barriers, in addition to pre-existing assumptions made by healthcare staff and services that the homogenous maternity population properly understands the benefits and timing of consuming folic acid, prior to conception [19, 20].

There is a dearth of evidence exploring similarities and differences in Pakistani, Bangladeshi and White British women’s attitudes, knowledge and perceptions of the association between folic acid consumption and reducing the risk of NTD, in addition to understanding healthcare professionals views [19]. This study addresses this gap; seeking to identify similarity and differences between the participant groups, to further understand this modifiable problem.

## Methods

### Aim

To review the similarities and differences in Pakistani, Bangladeshi and White British mothers health beliefs (attitudes, knowledge and perceptions) and health behaviour regarding their consumption of folic acid pre-conception, to reduce the risk of neural tube defects.

### Design

Our study used a descriptive qualitative research approach, implementing face-to-face focus group discussions with Pakistani, Bangladeshi or White British mothers (normal birth outcomes and mothers with poor birth outcomes) and semi-structured interviews or focus groups with service providers using semi-structured topic guides. This method is well suited for under researched areas where in-depth information is sought [21].

The findings in this paper form part of a larger mixed-methods study, which sought to identify similarities and differences in health beliefs and health behaviours of Pakistani, Bangladeshi and White British women, and how these contributed to perinatal mortality. The results and findings are published elsewhere [22, 23]. This paper concentrates reports on the findings of womens’ attitudes and experiences in using folic acid in the preconception period.

### Setting

Purposive sampling from Pakistani, Bangladeshi or White British mothers who had a normal birth outcome were recruited from local Children’s Centres to participate in focus group discussions [24]. They were eligible to take part if they had delivered a live-born baby after 37 weeks of gestation, in the preceding 6 to 24 months, weighing 2500 g and resided within a specified postcode area. Luton is a town with high rates of deprivation [25]. RG and NA held discussions with Children’s Centre managers and representatives from the local Luton community requested non-English speaking mothers were included in the study, as it was believed that non-English speaking mothers maternity experiences is disadvantaged; Therefore women were eligible to take part even if they did not speak English. Exclusions included women who had delivered infants before 37 weeks of gestation, outside the date range, who did not reside within the specified postcode area and his baby was under 2500 g, women from other ethnic groups and presence of gynaecological cancer or women who conceived through assistive reproduction techniques [26]. Recruitment methods included face-to-face invitation, poster advert and snowballing. The focus groups took place in community centres and church halls, in the proximity of the children centres from where they were recruited.

The second sample consisted of Pakistani Bangladeshi and white British bereaved mothers, who were invited to participate in semi-structured face-to-face interviews. These included women who had suffered a perinatal mortality in the preceding 6 to 24 months, his baby was either delivered stillborn or was classified as a neonatal death (0–7 days), residing within a specific postcode area. Women were eligible to participate if they did not speak English. Exclusions were women whose infant death occurred outside the preceding 6–24 months range specified; they failed to reside in the postcode area stated, women from other ethnic groups and presence of gynaecological cancer or women who conceived through assistive reproduction techniques [26]. The recruitment method used a gatekeeper (senior midwife) at the Luton and Dunstable University Hospital NHS Foundation Trust, to protect patient confidentiality and minimise distress [27]. The hospital sent potential participants a recruitment pack with a reply paid opt-in or opt-out contact sheet. Non-English speaking women were also invited using translators, who informed them about the studys’ purpose. The bereaved mothers were given the choice of the venue before the interview as their own home, a room in the community centre or a room at the University.

The third sample consisted of healthcare professionals (midwives (*n* = 2), community midwives (*n* = 14), key workers (*n* = 4), student midwives (*n* = 2), practice nurses (*n* = 1), GP (*n* = 1), and Health Visitors (*n* = 1) who were working on the local maternity care pathway (i.e. services providing preconception, antenatal, antepartum and postpartum care) were invited by poster, verbal invitation or letter to take part in focus groups or semi-structured interviews. The interviews for focus groups were held in staff offices or training rooms.

Recruitment took place between December 2014 and March 2016. Before commencement of any focus groups or interviews, participants were provided written information sheet detailing the purpose and aims of the research (including dissemination of results), and were encouraged to discuss any queries regarding participation in this study, prior to providing informed and signed consent. and. RG (who is a female, mature, research student and registered nurse who undertook NHS’ ‘*Good Clinial Practice*’ training as a condition of the ethic approval for this study) conducted the majority of the interviews or focus groups. Prior to the FG/interview, RG did not know the participants and had only met (some) of them during initial face to face recruitment and to respond to any enquires regarding taking part in the research. Two groups were held in Urdu and these were conducted by RM (with RG present) and two were conducted in Slyheti conducted by RS, (with RG present). The Focus groups or interviews were audio recorded and lasted between 62 and 132 min. Recruitment ceased when data saturation occurred and no new themes emerged from the transcripts [28].

Ethics approval was provided by University of Bedfordshire Institute for Health Research, (IHRREC442, November 2014) and NHS ethics committee (15/EE/0181: 157751, June 2015). Funding **-** The Steel Trust has provided funding to the University of Bedfordshire for RG to undertake research at Institute for Health Research, University of Bedfordshire, under the direction of NA and GR. The funders have no involvement in the research or publication.

### Materials

The topic guides for the focus groups and semi-structured interviews were developed in the same way for each participant group and were informed through a review of the existing literature and by the research aim (see Additional files 1 and 2 for copies of the topic guides). Each one built on existing themes in the literature that could be utilised flexibly during the focus group, interview and analysis stage, with probes that were grounded within the current evidence base. Several iterations were produced and piloted, prior to the final drafts. Piloting is a valuable method to ascertain whether the interview or focus group tool constrains participants and facilitates discussions on the intended topics [29]. The main themes of the topic guides are shown in Table 1.

**Table 1:** Main themes for each topic guide, by participant group

Mothers with a normal birth outcome	Bereaved mothers	Healthcare professionals
Perceptions of pregnancy	Rapport building	Information about maternity services
Knowledge and information	Mothers story, background and context	Using maternity services
Views on low birthweight, still birth and infant mortality	Mothers experience of maternity services and healthcare professionals	Providing services to diverse ethnic groups
Experiences of current services and maternity healthcare professionals	Knowledge and information	Views on high-risk pregnancies
Service improvements	Views on stillbirth	Service improvements
	Service improvements	

A bio-questionnaire was utilised with mothers who had a normal birth outcome and healthcare professionals to record demographic data, while bereaved mothers were asked these questions on commencement of the recorded interview, forming part of the rapport building.

### Data analysis

The demographic data from the bio-questionnaires and interview transcripts for all participant groups (normal birth outcome, bereaved mothers and healthcare professionals) was entered into Microsoft Excel to determine participant characteristics. RG produced verbatim transcripts for the English speaking focus groups or interviews; however, for the Urdu and Slyheti focus groups or interviews a university approved translator translated. Also, a second translator back-translated to ensure consistency of transliteration [30, 31]. NA reviewed the codes, themes and sub-themes until consistency was achieved between RG and NA. Additionally, four transcripts were presented back to participants for further verification of the accuracy of the transcript, ensuring trustworthiness was achieved [31–33]. Framework analysis is considered an appropriate method of analysing large amounts of qualitative data consequently is efficient in dealing with large amounts of data and contributes to the reliability and trustworthiness of the findings [29, 34]. RG and NA read the transcripts many times to become submerged and familiar with the narrative, this then allowed emergent themes to become evident, in addition to pre-existing themes from the topic guide. Next, the data was indexed and charted, using Microsoft Excel [35], which allowed the data to be organised and interpreted [34, 36].

## Results

The characterisitcs of the focus groups and interviews are shown in Table 2. The interviews with the six bereaved mothers were all conducted in participants’ homes. All the bereaved mothers had experienced a stillbirth and five out of six bereaved mothers were born and grew up in the UK, while the remaining Pakistani mother had been settled in the UK for 15 years. None of the mothers (normal birth outcome or bereaved) had smoked or consumed alcohol during their pregnancy. A number of themes and subthemes emerged from the narrative, which is organised by ‘mothers’ (i.e. normal birth outcome and bereaved mothers) and ‘healthcare professionals’.

**Table 2:** Focus group or interview participant characterisitcs

Stratification	Focus group/interview	Participants (N)	Lanugage of FG/interview	Ethicity	Duration of FG/interview	Lowest education level	Highest education level	Age range
Mothers with normal birth outcome	FG1	5	English	WB	70	GCSE	NVQ – level 2	23–42
	FG2	5	English	WB	69	GCSE	A Level	25–40
	FG3	4	English	Pakistani	84	NVQ level 2	Masters	28–38
	FG4	6	English	Pakistani	134	BSc	MBA	27–40
	FG5	4	English	Bangladeshi	73	BSc	MSc	24–39
	FG6	3	English	Pakistani	88	A level	MSc	32–41
	FG7	4	Slyheti	Bangladeshi	81	GCSE	BSc	26–32
	FG8	8	Slyheti	Bangladeshi	64	GCSE	BA (hons)	26–42
	FG9	7	Urdu	Pakistani	62	Year 8	MPA	23–49
Bereaved mothers	B1	1	English	WB	135		B-Tec	
	B2	1	English	Pakisani	49		A level	
	B3	1	English	Pakistani	32		GCSE	
	B4	1	English	Pakistani	40		A level	
	B5	1	English	WB	124		A level	
	B6	1	English	Bangladeshi	102		B-Tec	
	Focus group/interview	Participants (N)	Staff role	Ethnicty				
Health care professionals	FG1	2	Hospital Midwives	WB				
	FG2	10	Community midwives	WB				
	FG3	9	Community midwives,	WB, Pakistani				
	Interview 1	1	Health visitor	WB				
	Interview 2	1	Practice Nurse	Black African				
	Interview 3	1	GP	Pakistani				

### Findings from mothers

#### Limited knowledge on risk factors associated with adverse outcomes

The mothers discussed their limited knowledge of pregnancy and understanding of risks associated with causes of infant death. While the consumption of folic acid during pregnancy emerged from the narrative of all focus groups, it was evident that very few mothers were aware of the importance of taking folic acid before conception. This is seen in the following narratives;

Well you hear in the media that taking folic acid, that is not just the first 12 weeks but they recommend that you take it 3 months prior to conception, as well - so if you are planning, so I had been taking folic acid for 12 months before falling pregnant this time (White British mother).

Vitamins, folic acid they say is very important - important for the baby’s mental development. Before conceiving and after, we should continue taking it, as the mother’s and baby’s body becomes stronger (Bangladeshi mother).

When I went to the doctors, they told me that when I was trying… To take folic acid so I did throughout my pregnancy (Bangladeshi mother).

Conversely, the majority of mothers explained that they only commenced taking folic acid after receiving explicit advice from their GP or midwife, during their first appointment with the health professional.

You find out, as soon as you register yourself really, that is when they prescribe it [folic acid] when you take it is the first three months isn’t it? (Bangladeshi mother).

I started taking it [folic acid] as soon as I found out I was pregnant. I spoke to the midwife, and they gave me like a prescription to go and get erm, folic acid (White British mother).

The narratives revealed that mothers, regardless of their ethnicity were uncertain about the benefits of taking folic acid during the antenatal period. Discussions centred on ideas that pregnant women should not be taking ‘medication’ during pregnancy, or whether the folic acid was ‘pure’ enough for pregnant women. These ideas are evident in the following narratives:

I was a bit scared taking folic acid tablets and all the tablets for pregnancy; I was a bit scared of that, just in case like, I dunno, like there are all these scary (White British mother).

I think, psychologically I can’t take any medication when I’m pregnant I just feel it is better for the baby (White British mother).

The other thing with science, my sister-in-law is expecting, and my brother-in-law is really into his science stuff, and he doesn’t agree with folic acid because he says it’s not pure enough he said it’s got folate or something, or something else which I don’t know about, anyway but that is the thing, it depends on how deeply you go into things and you always find alternatives and things that are better but you have to read up on it don’t you? (Pakistani mother).

#### Mothers’ awareness of genetic risk factors

The mothers discussed their understanding of genetic influences and risks of congenital anomalies on adverse birth outcomes. Pakistani, Bangladeshi and WB participants spoke specifically about how they believed Downs Syndrome was heritable, although they did not discuss NTD, or any extra chromosomes. The following extracts show this;

It’s [Downs Syndrome] in your genes as well, isn’t it? (Pakistani mother).

I have got, like…. Back in Bangladesh, like quite a few members of my family are disabled and they have Down syndrome, and all of that, so you do… You do think it could become passed down (Bangladeshi mother).

Interestingly, adverse risks associated with genetic factors was discussed by two Pakistani mothers, but was in relation to consanguineous marriage and not folic acid. This is evident from the following narrative;

Lots of people saying that [cousin marriage increases risk of genetic problems] to be honest, but we are cousin married as well (Pakistani mother).

I think they [cousins] have more genetic problems, don't they? (Pakistani mother).

#### Barriers to consuming folic acid

The mothers’ narrative revealed several barriers to preconception consumption of folic acid. Several mothers explained the reasons why they did not consider taking folic acid at all, which included a lack of information;

It [folic acid] is not something that example a 16-year-old would even think about; I don’t think there’s that much information out there (Pakistani mother).

In addition, the mothers demonstrated misunderstandings of the causes of adverse birth outcomes and they showed limited understanding of the benefit of folic acid supplementation;

Because my mum took folic acid and she lost her baby so you can’t really say can you? Do you know I mean? There is people that take it and they have problems (White British mother).

No, I mean I could get the vouchers for it [folic acid] because I get the appropriate benefits, but I just never, I didn’t really know how to do it or how to apply for it. So I just didn’t bother it seemed pointless (White British mother).

This is seen in the following;

For me I didn’t know [I was pregnant], I had a fall, went to the hospital. They asked me if I was pregnant I said I don’t know if I had missed my periods… I said I don’t know. They checked – I was pregnant! My mum had figured it out by me being reluctant to eat certain foods. I was happy but felt embarrassed too (Bangladeshi mother).

One mother described consuming Marmite as an alternative to tablets when she forgot her tablet;

My step-dad was looking up on the Marmite, and you know that it said folic acid on there and erm, so I was a bit scared…so then I was taking it, and then if I forgot to take a tablet, I’d have a bit of marmite. (White British mother).

### Findings from healthcare professionals

#### Staff perceptions of mothers awareness of risk factors for NTD

The analysis revealed that there were conflicting opinions between healthcare professionals whether local mothers actually consumed folic acid *before* conception; some participants did not think the mothers did, as seen in the following:

It’s interesting because not lots of them take the folic acid, though …(White British).

Preconceptually? – no [folic acid] … (White British mother).

Conversely, other healthcare professionals believed that the majority of pregnant women did consume folic acid, prior to conception:

Yes, most people have [taken folic acid] (White British mother).

I think that they do, [take folic acid] – I mean from the diabetic perspective I am aware that we need to prescribe it because they are meant to have a high dose of folic acid … I think that that awareness is there (Practice Nurse, White British).

It was evident that health care professionals were aware of the confusion some mothers experience in respect of consuming folic acid in the early weeks. This is seen in the following extract;

…but the folic acid they have that in the first few weeks, most of the time they would have had it before we go in, but when I’m doing my assessment. I go through that, I say, ‘have you had you folic acid?’ some of them don’t remember what they’ve had, they having their pregnancy vitamin (HV1).

#### Using maternity services

Furthermore, the Practice Nurse reported that she believed local South Asian women had a good understanding of preconception care, because of delivering a previous baby;

I think that is quite likely, [taking folic acid] because …I think that a lot of the Asian ladies that we see it is not always the first child or they are not necessarily first generation Asian that are over here so basically they are British Asian as they were born here… so therefore the Asians may have a good concept of the services that are provided by the NHS (Practice Nurse, White British).

The community midwives explained that eligible mothers could receive vitamin and folic acid supplements; this is seen in the following;

No not everyone. Just the healthy start. They get the vitamin C and folic acid - is all in the healthy start (MW).

## Discussion

### Main findings

The majority of the findings in the study identified similarities in the mothers’ understanding of the benefits of folic acid consumption, irrespective of the mothers’ ethnicity or birth outcome. This study identified that very few women consumed folic acid before conception, nor did they understand the benefits of consumption, which would otherwise prevent congenital anomalies, including NTD in the early weeks of meiosis [16]. Moreover, only a few mothers described alternate sources of folic acid through alternate foods, such as leafy vegetables, which is commensurate with the findings of Yeasmin and Regmi [37].

Conversely, this study found that the majority of healthcare staff believed most women did commence folic acid consumption prior to conception, showing an important mismatch between women’s actual health behaviour in consuming folic acid, contrasted with health professionals perception of women’s health behaviour and subsequent understanding regarding pregnant women’s risk reduction for NTD. Importantly, this study also highlights that the majority of women consider Downs Syndrome (specifically) when discussing genetic factors, and fail to mention risks associated with NTD (e.g. spina bifida), suggesting a poor understanding of genetic factors and a need for increased public health awareness.

The common explanation for higher rates of congenital anomalies in Pakistani infants is consanguinity [38–40]. Interestingly, this study did highlight that Pakistani mothers associated congenital anomalies with consanguineous unions, as opposed to other known risk factors for congenital anomalies such as poor nutrient intake [41, 42], (including folic acid), diabetes and obesity [43–45]. Moreover, consanguinity contributes to autosomal recessive disorders, whereas NTDs are related to suboptimal folic acid consumption, although few studies make this distinction, which may obscure the precise aetiology behind congenital anomalies [46].

Current estimates suggest less than a third of women consume folic acid prior to conception [11, 13, 14], and the last decade has shown a trend in declining figures of consumption [3], meanwhile increasing trends have been observed in the prevalence of NTD [2]. This indicates more reseach is required to understand the reasons behind this pnenomenon better, and this study contributes to this necessary body of knowledge.

### Strengths and limitations

This study has a number of limitations. Luton is a deprived town and the findings may reflect lower educational levels as shown in the demographic profile of the town. More research is needed to determine an accurate prevalence rate for Luton, since this finding was obtained through a small number of self-reports and will identify the scope of the problem. There were also a number of strengths. This is the first study to identify similarities and differences between Pakistani, Bangladeshi and White British mothers understanding of consuming folic acid. In addition, much time and attention was given to recruiting representative women from the local community, including non-English speaking mothers. This process was time-consuming and involved the kind support and collaboration of many members of the community, Children’s Centres and local NHS Trust in supporting the focus groups and ensuring women’s experiences were documented. Consequently, since several distinct samples were used, this has facilitated triangulation of the data which increases the trustworthiness of the converged findings [47].

### Interpretation

Taken together, it appears that there is a need for increasing health literacy on the benefits of folic acid consumption (including the timing of commencing supplementation prior to pregnancy) for women regardless of ethnicity or ability to speak English which is perhaps best aimed at teenagers in formal educational environments, before they become pregnant.

## Conclusion

Few Pakistani, Bangladeshi or WB women understood the benefit of taking folic acid for optimising meiosis and reducing risks associated with NTD, while staff wrongly believed mothers supplement folic acid during the recommended preconception period. There is clearly a need to increase public health awareness, regarding the optimal time and benefits of consuming folic acid.

## Additional files

Additional file 1: Topic guide for lay mothers. This topic guide was the final approved version, after pilot revisions were made and used with mothers in focus groups, stratified by ethnicity and who had a normal birth outcome (see methods section for further description). (DOCX 58 kb)

Additional file 2: Topic guide for bereaved mothers. This topic guide was used with mothers who suffered a perinatal bereavement in face-to-face interviews (see Method section for a full description). (DOCX 45 kb).

## Abbreviations

FG: Focus group
NA: Nasreen Ali
NHS: National Health Service
NTD: Neural tube defect
P: Participnat
RG: Rebecca Garcia
RM: Riffat Mahmood
RS: Rukia Saalem
UK: United Kingdom
