# Supplementary material for: Understanding the consumption of folic acid during preconception, among Pakistani, Bangladeshi and white British mothers in Luton, UK: a qualitative study
Source: BMC Pregnancy Childbirth. 2018 Jun 15;18:234. doi: 10.1186/s12884-018-1884-0 (PMC6003022; doi:10.1186/s12884-018-1884-0)
Supplement: Supplementary file 1 — Topic guide for lay mothers. This topic guide was the final approved version, after pilot revisions were made and used with mothers in focus groups, stratified by ethnicity and who had a normal birth outcome (see methods section for further description). (DOCX 58 kb) [file 12884_2018_1884_MOESM1_ESM.docx]

#
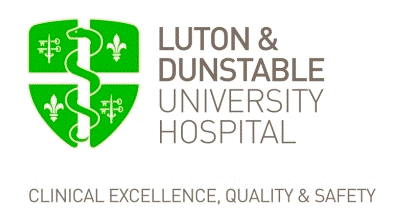

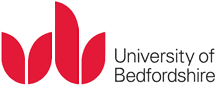
Explaining factors that contribute to low birth weight, stillbirth and infant mortality in Pakistani and Bangladeshi women living in Luton

| **Topic Guide – Bereaved Mothers** |
| --- |

1. **Introduction**

- Thank you for agreeing to take part.
- Give background & purpose to study: we want to identify factors that may contribute to low birth weight, still birth and infant mortality in babies of Pakistani and Bangladeshi women in Luton. This will help identify areas to improve maternity services in the future.
- Acknowledge the loss of their baby.
- Acknowledge that the topic is upsetting / sensitive. If they want to have a break at any time (and stop the recording) that is absolutely fine.
- Check what support is in place for after the interview/later that day (i.e. who), signpost to counselling services (give contact numbers).
- Explain the consent procedure, right to withdraw, confidentiality and audio recording of the discussion. Break at any time if required. Interview discussion to last 60-90 minutes. Check that they have understood the information sheet, confidentiality information and check understanding.
- Explain how the discussion that is going to take place will be used in the research.
- Findings form part of PhD thesis and will be published in academic journals and findings feedback local presentation to local service providers and interested community members.
- Complete consent forms, bio questionnaire.

**Rapport building** *[build on answers to establish rapport]*

How do you self-define you ethnic group?

*Probe: Pakistani, Kashmiri, Bangladeshi, Asian, Asian British, Muslim, English*

Have you lived in UK long?

*Probe: since birth or migrated when?*

Where did you go to school?

*Probe: location (UK or abroad), level of education attained.*

Tell me about who lives at home with you?

*Probe: for husband/partner/children/parents/parents-in-law.*

Do you work?

*Probe: for home-maker, house wife, carer, employed work, if employed what work.*

For the purposes of completeness, can I ask whether you smoked or drank alcohol during your pregnancy?

*Probe: how much/how often.*

1. **The mothers story and context**

*I would really like to start by hearing about you and your and experience. Is that okay?*

When did you first realise that something may not be right with your pregnancy?

- *Probe: for thoughts on your pregnancy, what signs suggested something was not right, e.g. decreased foetal movements, vaginal bleeding/discharge, stomach cramps, swollen ankles, “feelings something was wrong”.*
- *Probe: for views on when in the pregnancy did something go wrong? (first pregnancy, second pregnancy etc).*
- *Probe: what actions you took when you realised something was wrong e.g. friends, family, GP, A&E, Midwife, Obstetric ward, NHS Direct/111*
- *Probe: for views on what actions others took? Partner, family, GP, hospital staff.*
- *probe for: any actions taken or not taken that are important to you in respect of your culture? What support did you receive?*
- *Probe: for what happened next e.g. the delivery, what follow up received?*
- *Probe: what aftercare and/or counselling support did you access/receive (if not, why not, views on counselling support if received.*

I would like to hear your views on what you think went wrong.

- *Probe: for thoughts on why things went wrong in the pregnancy (personal health behaviours, access to care, help seeking behaviours, co-morbid factors, Gods will, genetic/hereditary factors or family history.*
- *Probe: for thoughts on the reasons behind decisions made (e.g. screening); autonomous or collaborative decision making e.g. Husband, family decision, doctors’ advice and why?*
- *Probe: for services accessed, post morte, MRI (if not why not).*

1. **Your experiences of maternity services and maternity healthcare professionals.**

*I’d like to ask about the issues related to your experience of maternity services and maternity healthcare professionals in Luton.*

How were you referred to maternity services in Luton at the beginning of the pregnancy?

- *Probe: for referral route, how referred*

*Probe: for how many weeks gestation at referral and factors influencing decision.*

What maternity services did you access?

- Probe: *for views on* *maternity services – lifestyle changes; preconception (preconception advice from GP), antenatal (booking, screening tests, specialist screening services, surveillance, antenatal class, birth preparation), specialist clinics (diabetes or hypertension), bereavement follow up (consultant or GP), bereavement midwife.*

I would like to ask you your views and experience of antenatal tests and checks (i.e. screening) services?

- *Probe: for experience of when in pregnancy, by whom, and if known why e.g. cousin marriage, hereditary risk.*
- *Probe: for experience and awareness of screening services/conditions (Downs syndrome, Thalassemia, normal growth/development and infectious diseases)*
- *Probe: for what information was provided, understanding of opting in or out of screening services, sufficient information to make confident decisions regarding pregnancy and risks/benefits of these decisions and impact of information on future pregnancies.*
- *Probe: for what views on decisions made e.g. screening vs. no screening, reasons for decision?*
- *Probe: for your views of religious reasons (perceptions of Fatwa), Gods will.*

In your view are there any reasons why you (or other Pakistani/Bangladeshi/White –*delete as appropriate*) women) in Luton wouldn’t use the local maternity services?

- *Probe: for views on favouring alternative healthcare (herbalists, traditional healers), pregnancy being a natural event and therefore not needing medical intervention, concealment/secrecy of pregnancy, lack of information of available services, unaware of benefits of services, fear of advice (termination of pregnancy), conflicts with cultural and religious beliefs, transportation problems, lack of confidence and trust in the service providers e.g. receiving misinformation/inadequate, biased or stereotyped information, previous experience of self or others poor outcomes, language and communication problems, lack of female staff/separate facilities for women, lack of understanding toward cultural and religious choices*

**3. Knowledge & information**

*I’d like to discuss with you what you know about the services that are offered in Luton for pregnant women and where you got your information and advice about your pregnancy from.*

What does the Luton maternity service offer to local women?

- *Probe: for views on awareness of available services at preconception (preconception advice from GP, folic acid, healthy eating), antenatal (booking, screening tests, surveillance, antenatal class, birth preparation), follow-up afterwards (consultant, GP)*
- *Probe: for views on who provided this information friends, family, Internet, television, radio (which stations) GP, midwives, health visitors, others (identify).*
- *Probe: for your ideas and perceptions of caring for yourself during pregnancy-what to do and what not to do. Medication during pregnancy verses allopathic or traditional medicine. Nutrition (eating down, vitamins). Work, rest.*
- *Probe: for views on whether information provided was enough to prepare you for the pregnancy and if not why not, cultural perceptions of the services.*

Was the information provided accessible and acceptable?

- *Probe: for views on what form the information was provided (oral, written).*
- *Probe: for views on whether the information understandable, any communication issues like language/literacy, if interpreters were available to ease communication, where they used.*
- *Probe: views on whether any part of the service impacted on patients cultural and religious values e.g. availability of female staff, screening advice, termination of pregnancies, informed choice.*
- *Probe: for information on autonomous/collaborative/guided decision making and what was important during this process.*
- *Probe: preparations for burial, washing, dressing, prayers materials.*

**4. Views on low birthweight, stillbirths and infant mortality**

*I would like to talk about your views on …[select appropriate: low birthweight or stillbirth or infant mortality].*

**Use only the appropriate section(s) below to reflect the mothers’ bereavement story:*

Low birth weight (pre-term deliveries or small for gestational age)

What do you understand is low birthweight?

- *Probe: views on reasons birthweight less than 2500 g or 5.5lb, small babies=healthy/unhealthy, growth restricted.*

What are the risk factors for low birthweight?

- *Probe: views on biological factors – perceptions about the causes of poor birth outcomes: diet and nutrition, late booking, comorbidities, , family genetic/hereditary history, cousin marriage, age, previous complications, ethnicity, known risk factors, pre-term birth (before 37 weeks) God’s will.*
- *Probe: views on cultural/religious perceptions: black magic (wind, curses), fatalistic explanations/Gods will:*

*.*

How can low birth weight be prevented, if at all?

- *Probe: views on adequate nutrition (Vitamin D, folic acid, iron, hot/cold foods, avoiding shellfish and high infection risk foods), regular ante-natal monitoring, ante-natal classes (education) termination of abnormal foetus (detected through screening), screening uptake, –screening for family genetic/hereditary history, cousin marriage ,*
- *Probe: views on cultural/religious perceptions avoiding cousin marriage, not smoking (including smokeless tobacco and betal nut) supernatural beliefs (avoiding the evening wind, curses, avoiding solar/lunar eclipses, amulets, enchanted water, prayer).*

Stillbirth

What are the risk factors for stillbirth?

- *Probe: views on reasons - biological factors – perceptions about the causes of poor birth outcomes: diet and nutrition, late booking, comorbidities, avoidance of cousin marriage, , age, previous complications, ethnicity, known risk factors, God’s will.*
- *Probe: views on cultural/religious perceptions: black magic, fatalistic explanations/Gods will:*

How can stillbirth be prevented, if at all?

- *Probe: views on adequate nutrition (Vitamin D, hot/cold foods, avoiding shellfish and high infection risk foods, folic acid, iron), regular ante-natal monitoring, ante-natal classes (education) termination of abnormal foetus (detected through screening), screening uptake,*
- *Probe: views on cultural/religious perceptions - avoidance of cousin marriage, not smoking (including smokeless tobacco and betal nut) supernatural beliefs (avoiding the evening wind, curses, avoiding solar/lunar eclipses, amulets, enchanted water, prayer).*

Infant death

What are the risk factors for infant death?

- *Probe: views on reasons biological factors – perceptions about the causes of poor birth outcomes: diet and nutrition, late booking, comorbidities, avoidance of cousin marriage, age, previous complications, ethnicity, known risk factors, God’s will.*
- *Probe: views on cultural/religious perceptions: black magic, fatalistic explanations/Gods will:*

How can infant death be prevented, if at all?

- *Probe: views on adequate nutrition (Vitamin D, hot/cold foods, avoiding shellfish and high infection risk foods, folic acid, iron), regular ante-natal monitoring, ante-natal classes (education) termination of abnormal foetus (detected through screening), screening uptake,*
- *Probe: views on cultural/religious perceptions - avoidance of cousin marriage, not smoking (including smokeless tobacco and betal nut) supernatural beliefs (avoiding the evening wind, curses, avoiding solar/lunar eclipses, amulets, enchanted water, prayer).*
- *Probe: views on breastfeeding (discarding colostrum), safe sleeping practices.*

**5. Service improvements**

*I would like to ask you your views on how services might be changed.*

Based on your experience, what are your views on how professional maternity staffin Luton might better meet your individual needs?

- *Probe: for advocates (or patient representative), female staff, female only antenatal classes, separate facilities for women.*
- *Probe: for culturally competent staff e.g. staff that have awareness of diverse religious, cultural beliefs, and different social needs (connectedness with others, friends, community) ,maternity services that empower and meet diverse needs, non-judgmental, and encourage inclusion and trust.*
- *Probe: for culturally appropriate services, i.e. how can things be done more culturally or religiously sensitive?*

What else could have been done to help you through your difficult time?

- *Probe: institutional support: for views on counselling and support services offered,*
- *Probe: professional support (staff) i.e. rapport, respect, empathy, listening,*
- *Probe: social support (partner, family, friends) i.e. respect, empathy, listening, acknowledgement*

If there was one recommendation you could make what would it be?

- Probe: views on how services could be improved to support bereaved mothers

**6. Closing. Any other comments, suggestions or questions**

*I would like to ask you for your final thoughts reflections as we come to the end of our discussion.*

*You have been through a difficult experience and I am grateful that you have shared it. Your views are very valuable to us and we hope that you have not found it too distressing to share your experience. Your views will help develop future maternity, as together with other women’s experiences, we will be able to highlight areas for change to make improvements for mothers and their families.*

*Ask if they would like summary of the findings of the research?*

Summarise key points of discussion

**[signpost support services]**
