# Supplementary material for: Understanding the consumption of folic acid during preconception, among Pakistani, Bangladeshi and white British mothers in Luton, UK: a qualitative study
Source: BMC Pregnancy Childbirth. 2018 Jun 15;18:234. doi: 10.1186/s12884-018-1884-0 (PMC6003022; doi:10.1186/s12884-018-1884-0)
Supplement: Supplementary file 2 — Topic guide for bereaved mothers. This topic guide was used with mothers who suffered a perinatal bereavement in face-to-face interviews (see Method section for a full description). (DOCX 45 kb). [file 12884_2018_1884_MOESM2_ESM.docx]

**
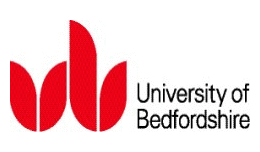
Identifying factors that contribute to low birth weight, stillbirth and infant mortality in Pakistani and Bangladeshi women living in Luton.**

| **Topic Guide – Lay Mothers** |
| --- |

1. **Introduction**

- Thanks.
- Introduce self, supervisors and University of Bedfordshire.
- Give background & purpose to study: we want to identify factors that may contribute to poor birth outcomes in babies of Pakistani and Bangladeshi women in Luton. This will help identify areas to improve maternity services in the future.
- Explain the idea of maternal health beliefs, how these are different in different cultures and how this as yet has not been properly explored in Pakistani or Bangladeshi women living in UK.
- Refer participants to information sheet – check that they have read and understood the information sheet, confidentiality information and check understanding.
- Explain the consent procedure, right to withdraw, confidentiality and audio recording of the discussion. Break at any time if required. Interview discussion to last 60-90 minutes.
- Explain how the discussion that is going to take place will be used in the research.
- Findings form part of PhD thesis and will be published in academic journals and findings feedback local presentation to local service providers and interested community members.
- Respectful listening.
- Allow for questions.
- Complete consent forms, bio questionnaire and conduct icebreaker.

**2. Perceptions of pregnancy**

*I would like to ask you your views on pregnancy, before during and after birth.*

What have/had you heard about pregnancy before having your children?

- *Probe: perceptions of pregnancy-difficult/easy, risk factors associated with pregnancy (low birthweight stillbirths infant deaths, congenital abnormalities/disabilities), number of pregnancies.*
- *Probe: views on when to see a doctor (secrecy concealment shame confirmation of pregnancy, pregnancy - natural event (so medical confirmation not required) awareness of screening, awareness of the benefits of antenatal clinic.*

In your view/experience what are the symptoms of a healthy pregnancy and one where something may not be right with the pregnancy?

- *Probe: for what is considered a healthy pregnancy e.g. missed period, morning nausea and/or sickness, tiredness, sore breasts, breast changes (larger), changes in skin colour (e.g. darker nipples, changes to sense of smell or taste (food likes/dislikes, some weight gain.*
- *Probe: for pregnancy where something may not be right e.g. decreased foetal movements, vaginal bleeding/discharge, stomach cramps, swollen ankles.*

Where is this information from?

- *Probe: family friends Internet, television, radio (which stations), GPs midwives, health visitors, others (identify).*

Can you explain to me what being pregnant and giving birth to a healthy child means to you?

- *Probe: impact on status – own family, in-laws, extended family, community.*
- *Probe: impact on the role in own family and in-laws.*
- *Probe: cultural expectation to have children, a natural event, a natural life progression, special event.*
- *Probe: impact on perceptions of attractiveness, religiosity (being closer to God during pregnancy and birth).*

What roles do/did people you know play during your pregnancy?

- *Probe: husband, family, in-laws, friends, extended family, community.*
- *Probe: healthcare staff (GP, midwives, health visitors, hospital consultants).*

How do people you have mentioned influence decisions about your pregnancy and birth?

- *Probe: what decisions they influence e.g. getting pregnant, pregnancy choices (screening, delivery, diet and lifestyle choices) during pregnancy and birth.*
- *Probe: support (explore what type of support) and the role of support on autonomy, privacy and confidentiality.*
- *Probe: modern medicine verses allopathic or traditional medicine.*

**3. Knowledge & information**

*I’d like to discuss where you got your pregnancy advice and information from before, during and after pregnancy.*

What does the Luton maternity service offer to local women?

- *Probe: for awareness of available services at preconception (preconception advice from GP), antenatal (booking, screening tests, surveillance, antenatal class, birth preparation), postnatal (midwifery checks, mother and baby monitoring, baby clinics with health visitor, six-week check up with GP, family planning advice).*
- *Probe: who provided this information friends, family, Internet, television, radio (which stations) GP, midwives, health visitors, others (identify).*
- *Probe: if information provided was enough to prepare you for the birth, if not why not?*

Was the information provided accessible and acceptable?

- *Probe: what form was the information provided (oral, written).*
- *Probe: was the information understandable, any communication issues like language/literacy, if interpreters were available to ease communication, where they used.*
- *Probe: if any part of the service impacted on patients cultural and religious values e.g. availability of female staff, screening advice termination of pregnancies, informed choice.*

**4. Views on low birthweight, stillbirths and infant mortality**

*I would like to talk about your views on low birthweight, stillbirths and infant mortality.*

What do you understand is low birthweight?

- *Probe: birthweight less than 2500 g or 5.5lb, small babies=healthy/unhealthy*

What are the risk factors for low birthweight?

- *Probe: biological factors – perceptions about the causes of poor birth outcomes: diet and nutrition, late booking, comorbidities, consanguinity, age, previous complications, ethnicity, known risk factors, pre-term birth (before 37 weeks) God’s will.*
- *Probe: cultural/religious perceptions: black magic (wind, curses), fatalistic explanations/Gods will:*

How can low birth weight be prevented, if at all?

- *Probe: adequate nutrition (Vitamin D, folic acid, iron, hot/cold foods, avoiding shellfish and high infection risk foods), regular ante-natal monitoring, ante-natal classes (education) termination of abnormal foetus (detected through screening), screening uptake,*
- *Probe: cultural/religious perceptions - consanguineous marriages, not smoking (including smokeless tobacco and betal nut) supernatural beliefs (avoiding the evening wind, curses, evil eye, avoiding solar/lunar eclipses, amulets, enchanted water, prayer).*

What are the risk factors for stillbirth?

- *Probe: biological factors – perceptions about the causes of poor birth outcomes: diet and nutrition, late booking, comorbidities, consanguinity, age, previous complications, ethnicity, known risk factors, God’s will.*
- *Probe: cultural/religious perceptions: black magic, fatalistic explanations/Gods will:*

How can stillbirth be prevented, if at all?

- *Probe: adequate nutrition (Vitamin D, hot/cold foods, avoiding shellfish and high infection risk foods, folic acid, iron), regular ante-natal monitoring, ante-natal classes (education) termination of abnormal foetus (detected through screening), screening uptake,*
- *Probe: cultural/religious perceptions - consanguineous marriages, not smoking (including smokeless tobacco and betal nut) supernatural beliefs (avoiding the evening wind, curses, evil eye, avoiding solar/lunar eclipses, amulets, enchanted water, prayer).*

What are the risk factors for infant death?

- *Probe: biological factors – perceptions about the causes of poor birth outcomes: diet and nutrition, late booking, comorbidities, consanguinity, age, previous complications, ethnicity, known risk factors, God’s will.*
- *Probe: cultural/religious perceptions: black magic, fatalistic explanations/Gods will:*

How can infant death be prevented, if at all?

- *Probe: adequate nutrition (Vitamin D, hot/cold foods, avoiding shellfish and high infection risk foods, folic acid, iron), regular ante-natal monitoring, ante-natal classes (education) termination of abnormal foetus (detected through screening), screening uptake,*
- *Probe: cultural/religious perceptions - consanguineous marriages, not smoking (including smokeless tobacco and betal nut) supernatural beliefs (avoiding the evening wind, curses, evil eye, avoiding solar/lunar eclipses, amulets, enchanted water, prayer).*
- *Probe: breastfeeding (discarding colostrum), safe sleeping practices.*

**5. Your experiences of current services and maternity healthcare professionals**

*I’d like to ask about the issues related to current maternity services and maternity healthcare professionals in Luton.*

How were you referred to maternity services in Luton?

- *Probe: for referral route and experience, late booking (after 12 weeks of pregnancy*)

What maternity services have you or did you access?

- Probe: *maternity services - preconception (preconception advice from GP), antenatal (booking, screening tests, surveillance, antenatal class, birth preparation), postnatal (midwifery checks, mother and baby monitoring, baby clinics with health visitor, six-week check up with GP, family planning advice).*

Where you offered screening services?

- *Probe: awareness that screening services/conditions.*
- *Probe: for information provided, understanding of opting in or out and concept of informed choice, impact of information on future pregnancies.*

Are you aware of any reasons why women in Luton wouldn’t use the local maternity services?

- *Probe: favouring alternative healthcare (herbalists, traditional healers), pregnancy being a natural event – not needing medical intervention, concealment/secrecy of pregnancy, lack of information of available services, unaware of benefits of services, fear of advice (termination of pregnancy), transportation problems, lack of trust in the service providers, language and communication problems, lack of female staff/separate facilities for women, cultural and religious beliefs.*

How could maternity services in Luton be more suited to your needs?

- *Probe: advocates, female staff, female only antenatal classes, separate facilities for women, culturally competent staff, community-based support.*

**6. Closing. Any other comments, suggestions or questions**

Any further thoughts and reflections?

Summarise key points of discussion
